# A 20‐year study of suicide death in a statewide autism population

**DOI:** 10.1002/aur.2076

**Published:** 2019-01-21

**Authors:** Anne V. Kirby, Amanda V. Bakian, Yue Zhang, Deborah A. Bilder, Brooks R. Keeshin, Hilary Coon

**Affiliations:** ^1^ Department of Occupational and Recreational Therapies University of Utah Salt Lake City UT; ^2^ Department of Psychiatry University of Utah Salt Lake City UT; ^3^ Department of Internal Medicine University of Utah Salt Lake City UT; ^4^ Study Design and Biostatistics Center, Center for Clinical and Translational Science University of Utah Salt Lake City UT; ^5^ Department of Pediatrics University of Utah Salt Lake City UT; ^6^ Center for Safe and Healthy Families, Primary Children's Hospital Salt Lake City UT

**Keywords:** autism spectrum disorder, suicide, population, epidemiology, mental health

## Abstract

**Scientific Summary:**

Growing concern about suicide risk among individuals with autism spectrum disorder (ASD) necessitates population‐based research to determine rates in representative samples and to inform appropriate prevention efforts. This study used existing surveillance data in Utah to determine incidence of suicide among individuals with ASD over a 20‐year period, and to characterize those who died. Between 1998 and 2017, 49 individuals with ASD died by suicide. Suicide cumulative incidence rates did not significantly differ between 1998 and 2012 across the ASD and non‐ASD populations. Between 2013 and 2017, the cumulative incidence of suicide in the ASD population was 0.17%, which was significantly higher than in the non‐ASD population (0.11%; *P* < 0.05). During this period, this difference was driven by suicide among females with ASD; suicide risk in females with ASD was over three times higher than in females without ASD (relative risk (RR): 3.42; *P* < 0.01). Among the individuals with ASD who died by suicide, average age at death and manner of death did not differ significantly between males and females. Ages at death by suicide ranged from 14 to 70 years (*M*[SD] = 32.41[15.98]). Individuals with ASD were significantly less likely to use firearms as a method of suicide (adjusted odds ratio: 0.33; *P* < 0.001). Study results expand understanding of suicide risk in ASD and point to the need for additional population‐based research into suicide attempts and ideation, as well as exploration of additional risk factors. Findings also suggest a need for further study of female suicide risk in ASD. ***Autism Research** 2019, 12: 658–666*. © 2019 The Authors. *Autism Research published by International Society for Autism Research* published by Wiley Periodicals, Inc.

**Lay Summary:**

This study examined suicide risk among individuals with autism spectrum disorder (ASD) in Utah over a 20‐year period. Risk of suicide death in individuals with ASD was found to have increased over time and to be greater than in individuals without ASD between 2013 and 2017. Females with ASD were over three times as likely to die from suicide as females without ASD. Young people with ASD were at over twice the risk of suicide than young people without ASD. Individuals with ASD were less likely than others to die from firearm‐related suicides.

## Introduction

Suicide is a global public health crisis [WHO, 2018]. In the U.S., 12.93 per 100,000 individuals die by suicide per year, making it the tenth leading cause of death overall, and the second leading cause of death among young people ages 10–34 [CDC, 2016]. Suicide occurs more commonly among males than females at a ratio of 3.5 to 1, respectively [NIMH, 2018]. Women also tend to use less violent methods of suicide than males [Ajdacic‐Gross et al., 2008].

Families, clinicians, and autistic individuals have raised growing concern as emerging research suggests high rates of suicidal thoughts and behaviors among individuals with autism spectrum disorder (ASD) [Veenstra‐VanderWeele, 2018]. However, much of this research to date has focused on clinical or convenience samples reporting varying rates [Zahid & Upthegrove, 2017]; little population‐based data exists on risk of death by suicide in the ASD population. In this study, we focused specifically on suicide death among individuals with ASD in Utah to identify incidences and characteristics of the decedents. This type of population‐based research is needed to fully understand the problem of suicide in ASD and who is at risk while expanding on prior studies.

### *Suicidal Thoughts and Behaviors in ASD*

Review studies have reported wide ranging rates of suicidal thoughts and behaviors in ASD populations [e.g., Segers & Rawana, 2014; Zahid & Upthegrove, 2017]. Zahid and Upthegrove [2017] identified studies reporting a range of suicide attempts from 7% to 47% and suicidal ideation in up to 72% of cases in small samples. Cassidy et al. [2014] reported suicidal ideation in 66% of their clinical sample of 374 adult‐diagnosed individuals with ASD (specifically, Asperger's syndrome) in the United Kingdom. This rate exceeded that of the general population by over ninefold. Chen et al. [2017] recently published a population‐based study that examined suicide attempts among 5,618 Taiwanese adolescents and adults with ASD. These individuals with ASD experienced higher rates of suicide attempts when compared with 22,472 age‐ and sex‐matched controls (3.9% vs. 0.7%, respectively), even after accounting for relevant demographic characteristics and co‐occurring psychiatric diagnoses (hazard ratio = 6.07; 95% confidence interval (CI), 4.64–7.93) [Chen et al., 2017].

Because suicide is a relatively rare occurrence, and ASD affects approximately 2% of the population, some researchers have opted to examine relationships between suicidality and autistic traits within samples of individuals without an ASD diagnosis. For example, Culpin et al. [2018] recently identified a significant relationship between social communication impairments and suicidal intent among over 5,000 youth in the U.K. (adjusted RR = 2.14, *P* = 0.004). In a sample of 163 young adults in the U.K., Pelton and Cassidy [2017] also found that self‐reported autistic traits predicted suicidal behavior in mediation models, with significant indirect effects found through both perceived burdensomeness (*P* = 0.001) and thwarted belonging (*P* = 0.001).

### *Suicide Deaths in ASD*

Only one study, to our knowledge, has examined suicide death among individuals with ASD using population‐based data. This study, conducted in Sweden by Hirvikoski et al. [2016], used national health data to determine causes of mortality in ASD. Overall, 83 individuals with ASD in their sample (0.31%) died by suicide, compared with 0.04% of the general population—almost an eight‐fold difference [Hirvikoski et al., 2016]. They identified suicide as one of the top three most common causes of premature mortality among individuals with ASD without co‐occurring intellectual disability (ID); this group was over nine times as likely to die by suicide than those without ASD (OR: 9.40; 95% CI: 7.43–11.9). Individuals with ASD and ID also had higher rates than in the general population, but this risk was less pronounced (OR: 2.41; 95% CI: 1.14–5.11). Results regarding females with ASD completing suicide were notable. Because both suicide and ASD are less common among females when considered independently, one might expect suicide among females with ASD to be quite rare. However, in Hirvikoski et al.'s [2016] study, females with ASD were over 13 times more likely than females in the general population to die by suicide (OR: 13.05; 95% CI: 8.73–19.5).

### *Study Purpose*

Existing research on suicidal thoughts and behaviors have often relied on clinical or convenience samples. These approaches introduce methodological biases that can result in over‐ and under‐inflated reporting of suicidality in ASD. The single prior population‐based study of suicide death among individuals with ASD occurred in Sweden. In this study, we used existing population‐wide surveillance data in the U.S. state of Utah to calculate the incidence (in 5‐year intervals) of suicide deaths in people with ASD over a 20‐year period (1998 to 2017) in total, as well as by sex, and compared suicide risk in people with versus without ASD. Further, we examined death certificate data on cases who had an ASD diagnosis and died by suicide (i.e., ASD + suicide cases) including sex, race, death age, occupational status, marital status, and manner of death (i.e., specific suicide method), and compared these characteristics in males versus females. Finally, we compared characteristics of ASD + suicide cases with other suicide cases (i.e., non‐ASD + suicide cases). Based on existing research, we hypothesized that suicide incidence would be significantly higher in the ASD population than in the non‐ASD population. In particular, we anticipated that the incidence of suicide among females in the ASD population would be significantly higher than females in the non‐ASD population. We also hypothesized that violent methods of death would be more frequently used in males with ASD than females with ASD, and that the frequency of violent method of death use would be higher in individuals with ASD compared to those without ASD.

This is the first U.S. study to examine incidence of suicide death in a population‐based sample of individuals with and without ASD. By studying completed suicide (rather than suicidality) among individuals with an identified ASD diagnosis (rather than autistic traits) in a state population (rather than using clinical samples), we are able to reduce potential sources of bias and understand suicide risk in the U.S. context.

## Methods

This retrospective cohort study was approved by the Utah Registry of Autism and Developmental Disabilities (URADD) Oversight Committee and the Institutional Review Boards of the University of Utah, Utah Department of Health, and Resource for Genetic and Epidemiologic Research Review Committee, which is an oversight body that regulates Utah Population Database (UPDB) access. The period of time of the study was 1998 to 2017, observed in 5‐year intervals.

### *Data Sources*

Four sources of existing data were utilized for this study: URADD statewide autism surveillance data, statewide suicide surveillance data collected by the Utah Office of the Medical Examiner (OME), the UPDB, and Utah's Indicator‐Based Information System for Public Health (IBIS‐PH). URADD and OME data were linked with UPDB data internally by UPDB staff and identifiers were removed prior to analysis by the research team.

#### Utah registry of autism and developmental disabilities

URADD is a statewide registry of individuals with an ASD diagnosis. In Utah, ASD is a reportable health condition under Utah Health Code Chapter 26 Title 7 Section 4, which established URADD to conduct state and population‐wide ascertainment for ASD. ASD case status is determined by URADD based on the presence of a community‐based medical diagnosis of ASD; the electronic data warehouses of all major healthcare providers in Utah, including the Utah Department of Health, private/public clinics and hospitals, and behavioral health centers, are queried on a biannual basis to identify individuals with an ASD diagnostic code (i.e., ICD‐9 299.X or ICD‐10 F48.X). URADD data was linked to anonymous numeric case identifiers within the UPDB for this study. All data linking within the UPDB is secure, using encrypted servers. Although URADD also ascertains children with ASD based on an autism special education eligibility, the current ASD cohort were identified exclusively through the presence of an ASD diagnosis in medical records.

The URADD data used for this study included 16,904 (76% male, 24% female) total individuals with ASD alive at the beginning of 1998 and at least 5 years of age in 2013. The ages of those at least 5 years in 1998 (start of first time period) ranged from 5–89 (*M*(*SD*) = 18.29(14.48)), and in 2013 (start of final time period) ranged from 5–95 (*M*(*SD*) = 18.38(12.05)). Race was documented as 72.5% White, 24.5% Unknown, 1.5% Multiple Races, and < 1% for all other racial groups, and ethnicity was documented as 8% Hispanic, 39% not Hispanic, and 53% Unknown.

#### OME suicide dataset

The Utah OME has had a research partnership with the University of Utah for two decades, and maintains data on all suicide deaths in the state. Suicide determination is made by the state‐centralized Utah OME using consistent protocols involving review of detailed investigation of the scene of the death and circumstances of death, determination of medical conditions by full autopsy, review of medical and other public records concerning the case, interviews with survivors, and toxicology (drugs, alcohol, nicotine, etc.). Suicide determination is given conservatively due to its impact on survivors. As with URADD data, OME data from suicide cases were securely linked within the UPDB to allow matching with ASD case status for this study.

The OME data used for this study included 8,827 (78% male, 22% female) total individuals who died by suicide between 1998 and 2017. Their ages at death ranged from 7 to 97 (*M*(*SD*) = 41.8(17.3)). Race was documented as 92.3% White, 2.7% Multiple races, 2.2% Unknown, 1.2% American Indian or Alaska Native, and < 1% for all other racial groups, and ethnicity was documented as 8% Hispanic, 80% not Hispanic, and 12% Unknown.

#### Utah population database

The UPDB is a large, state‐wide database containing demographic, vital records, medical and genealogical data, and supports research on genetics, epidemiology, demography, and public health. The UPDB has been established for over 30 years and contains death certificate data since 1904. The UPDB can internally link to other compatible data sources, and has strict protocols in place to protect data security; the UPDB links to the OME and URADD datasets. For this study, birth and death certificate data were also securely linked. Study investigators were given deidentified data for analysis.

#### Utah's indicator‐based information system for public health

The final data resource accessed was whole population statistics for the state of Utah, available from IBIS‐PH (Retrieved 31 May 2018 and 5 December 2018 from the Utah Department of Health, Indicator‐Based Information System for Public Health Web site: http://ibis.health.utah.gov), including the population count of individuals over the age of 5 years by sex within four time periods (1998–2002; 2003–2007; 2008–2012; 2013–2017). We subtracted URADD sample sizes to generate non‐ASD population count. For this study, we did not have access to detailed demographic data on the entire population over the 20 year period.

### *Variables*

Variables included in this study from the URADD and OME suicide datasets were sex (male/female), birth date (month and year), death date (month and year), age at death (in years), marital status (never married, married, other [widowed, divorced, separated, other], missing or unknown), and occupational status (multiple categories collapsed into: student, employed, did not work, missing). Socioeconomic status was examined, but missing data on employment precluded inclusion. ASD case status was determined by inclusion in URADD. Suicide case status was determined by inclusion in the OME suicide dataset. Method of death information from death certificates was categorized two ways, first as violent or nonviolent and second as firearm‐related or not. As they have been conceptualized elsewhere [e.g., Stenbacka & Jokinen, 2015], methods considered to be violent included firearm/gunshot, hanging or strangulation, and blunt force injury (e.g., jumping from a high point, stepping in front of a train), and those considered non‐violent included poisoning by asphyxiants (e.g., carbon monoxide) and intoxication (e.g., drug overdose).

### *Data Analysis*

We used R, Version R.3.4.4, and the Statistical Package for Social Sciences (SPSS), Version 25, to conduct the data analysis. Children under the age of 5 at the beginning of each study interval were not included in the calculations. Cumulative incidences were calculated within five‐year intervals in the ASD and non‐ASD populations, overall and by sex. RR for suicide in the ASD versus the non‐ASD populations was calculated, overall and by sex; unadjusted RR was used because we did not have demographic data on the non‐ASD population on the individual level. Incidence rates, accounting for person‐time (years), were also calculated within five‐year intervals in the ASD population; we did not have sufficient data to complete this analysis in the non‐ASD population. Death ages for the ASD + suicide cases were examined by time period and sex. Chi‐squared and *t*‐tests were used to test for differences in case characteristics (i.e., sex, race, death age, marital status, occupational status, method of death) between males and females and between ASD + suicide cases and non‐ASD + suicide cases.

Based on our results, we conducted additional analyses to further understand study findings. First, due to the relatively young ages of suicide cases in the ASD population and the known issues related to diagnostic ascertainment of ASD over time, we calculated the 2013–2017 cumulative incidence focused specifically on young people (5–30 years of age at the beginning of the time period) in the ASD and non‐ASD populations. Second, we conducted a logistic regression analysis to determine if age and/or sex explained our findings related to suicide deaths involving firearms.

## Results

### *Incidence*

Cumulative incidences of suicide deaths in the ASD and non‐ASD populations during each five‐year time interval, along with the unadjusted RR, are displayed in Table 1. In the first 15 years of the study (1998–2012), we did not observe differences in suicide cumulative incidences between the ASD and non‐ASD populations. For the most recent time interval (2013–2017), the cumulative incidence of suicide death in the ASD population was 0.17%, which is significantly higher than the non‐ASD population cumulative incidence of 0.11% (RR: 1.57 [95% CI: 1.08–2.28]). All seven of the females in the ASD population who died by suicide had a death date in the most recent time interval (2013–2017). Between 2013 and 2017, the cumulative incidence for females in the ASD population was 0.17%, which is significantly higher than the female non‐ASD population cumulative incidence of 0.05% (RR: 3.43 [95% CI: 1.63–7.23]). We also directly compared female and male groups in the 2013–2017 time interval, finding that non‐ASD males were significantly more likely than non‐ASD females to die by suicide (RR: 3.15 [95% CI: 2.89–3.44]), but the RR of suicide did not differ between males and females with ASD (RR: 0.94 [95% CI: 0.40–2.20]). Incidence rates, accounting for person‐years, in the ASD population are displayed in Table 2. The observed pattern of increase over time is consistent with the cumulative incidence results. The highest incidence rate was observed in the 2013–2017 time interval, with an incidence rate of 0.32.

**Table 1:** Cumulative Incidences of Suicide in the ASD and Non‐ASD Populations in Five‐Year Intervals, Total and by Sex, and Unadjusted RR

	ASD		Non‐ASD		ASD vs. Non‐ASD Unadjusted Relative Risk (95% CI)
	Cases/Total	Incidence (95% CI)	Cases/Total	Incidence (95% CI)	
1998–2002	2 / 5,202	0.04% (0.01–0.15)	1,617/1,928,484	0.08% (0.08–0.09)	0.46 (0.11–1.83)
Male	2/3,750	0.05% (0.01–0.21)	1,300/957,653	0.14% (0.13–0.14)	0.39 (0.10–1.57)
Female	0/1,452	‐	317/970,831	0.03% (0.03–0.04)	–
2003–2007	5/8,722	0.06% (0.02–0.14)	1,841/2,126,249	0.09% (0.08–0.09)	0.66 (0.28–1.59)
Male	5/6,514	0.08% (0.03–0.19)	1,487/1,062,486	0.14% (0.13–0.15)	0.55 (0.23–1.32)
Female	0/2,208	–	354/1,063,763	0.03% (0.03–0.04)	–
2008–2012	14/13,890	0.10% (0.06–0.17)	2,529/2,391,862	0.11% (0.10–0.11)	0.95 (0.56–1.61)
Male	14/10,532	0.13% (0.08–0.23)	1,975/1,194,905	0.17% (0.16–0.17)	0.80 (0.48–1.36)
Female	0/3,358	–	554/1,196,957	0.05% (0.04–0.05)	–
2013–2017	28/16,907	0.17% (0.11–0.24)	2,791/2,630,221	0.11% (0.10–0.11)	1.56 (1.08–2.26)
Male	21/12,883	0.16% (0.10–0.25)	2,123/1,315,867	0.16% (0.15–0.17)	1.01 (0.66–1.55)
Female	7/4,023	0.17% (0.08–0.38)	668/1,314,355	0.05% (0.05–0.05)	3.42 (1.63–7.20)

CI = confidence interval. Incidence in the ASD group and relative risk were not calculated for females in the first three intervals because there were zero cases in the ASD group.

**Table 2:** Incidence Rates of Suicide in the ASD Population in Five‐Year Intervals, Total and by Sex

	Cases/Person‐Years	Incidence Rate (95% CI)
1998–2002	2/36,626	0.03 (0.007–0.109)
Male	2/27,076	0.04 (0.009–0.148)
Female	0/9,550	–
2003–2007	5/57,118	0.04 (0.018–0.105)
Male	5/42,872	0.06 (0.024–0.14)
Female	0/14,246	–
2008–2012	14/64,076	0.11 (0.065–0.184)
Male	14/48,269	0.15 (0.086–0.245)
Female	0/15,807	–
2013–2017	28/43,154	0.32 (0.224–0.47)
Male	21/32,220	0.33 (0.213–0.5)
Female	7/10,934	0.32 (0.153–0.671)

When looking specifically at young people at risk during the 2013–2017 time period, we identified a significantly higher cumulative incidence of suicide in females with vs. without ASD (0.15% [95% CI: 0.003–0.302] vs. 0.03% [95% CI: 0.026–0.035], RR: 5.07 [95% CI: 1.883–13.636]). In contrast, there not a significant difference in the cumulative incidence of suicide in males with vs. without ASD (0.17% [95% CI: 0.083–0.252] vs. 0.11% [95% CI: 0.083–0.252], RR: 1.56 [95% CI: 0.936–2.603]). When looking at the total population (males and females), the cumulative incidence of suicide among young people with ASD during 2013–2017 was significantly higher than in the non‐ASD population (0.16% [95% CI: 0.09–0.238] vs. 0.07% [95% CI: 0.065–0.074], RR: 2.38 [95% CI: 1.509–3.744]).

### *Case Characteristics*

In total, we identified 49 individuals with ASD who died by suicide during the 20‐year period. Table 3 contains description of case characteristics and sex differences. There were seven females (14%) and 42 males (86%). Regarding race, 44 (88%) of the cases were White, five (10%) were multiple races, and one (2%) was American Indian or Alaska native; none of the cases were known to be of Hispanic ethnicity (98% non‐Hispanic, 2% unknown). The cases closely reflect the racial demographics of the state (Utah residents are 90.9% White), though diverge on ethnicity (Utah residents are 14% Hispanic; U.S. Census Bureau, 2017). Males and females did not significantly differ regarding death age, marital status, or occupational status.

**Table 3:** ASD + Suicide Case Characteristics, Total and by Sex

	Total *N* = 49	Male *N* = 42	Female *N* = 7	Sex Differences (*χ* ^2^ or *t*‐test, *P*‐value)
Age at Death (years)				*t* = − 0.6, *P* = 0.55
Range	14–70	14–70	16–43	
Mean (SD)	32.41 (15.98)	32.85 (16.75)	29.72 (11.64)	
Median	27	26.40	29.96	
Marital Status				*χ* ^2^ = 5.3, *P* = 0.15
Married	6 (12%)	5 (12%)	1 (14%)	
Never married	24 (49%)	23 (55%)	1 (14%)	
Other	5 (10%)	3 (7%)	2 (29%)	
Missing	14 (29%)	11 (26%)	3 (43%)	
Occupation				*χ* ^2^ = 3.4, *P* = 0.33
Student	12 (24.5%)	11 (26.2%)	1 (14.3%)	
Employed	12 (24.5%)	11 (26.2%)	1 (14.3%)	
Did not work	5 (10.2%)	3 (7.1%)	2 (28.6%)	
Missing	20 (40.8%)	17 (40.5%)	3 (42.9%)	
Method of Suicide^a^				*χ* ^2^ = 0.6, *P* = 0.43
Violent	36 (73%)	30 (71%)	6 (85.7%)	
Nonviolent	13 (26%)	12 (29%)	1 (14.3%)	

a Methods of suicide considered to be violent included firearm/gunshot, hanging or strangulation, and blunt force injury (e.g., jumping from a high point, stepping in front of a train); methods considered to be nonviolent included poisoning by asphyxiants (e.g., carbon monoxide) and intoxication (e.g., drug overdose).

In comparison with non‐ASD + suicide cases, ASD + suicide cases had significantly younger average death ages (32.4 years vs. 41.8 years; *t* = −3.8, *P* < 0.001). When looking at death ages over time, we identified wider age spans in the second half of the observed period; the youngest (14 years) and oldest (70 years) cases died between 2008 and 2017 (see Table 4).

**Table 4:** Death Age by Time Period in ASD+Suicide Cases

	1998‐2002	2003‐2007	2008‐2012	2013‐2017	
	*N* = 2	*N* = 5	*N* = 14	*N* = 21	*N* = 7
	Male	Male	Male	Male	Female
Age at Death (years)					
Range	24.2–35.0	16.0–28.0	14.0–69.8	18.9–68.5	16.3–43.1
Mean (SD)	29.6 (5.4)	20.5 (4.2)	41.8 (21.3)	30.1 (11.9)	29.7 (10.8)
Median	29.6	19.0	39.2	26.7	30.0

*Note*. Females only present in final time period.

Males and females with ASD did not differ regarding method of suicide. Combined, 73% of the ASD + suicide cases used methods for suicide considered to be violent; the remaining 26% used nonviolent methods. ASD + suicide cases were equally likely as the non‐ASD + suicide group to use violent suicide methods (36/49 vs. 6,490/8,414; *χ* ^2^ = 0.37, *P* = 0.54), but significantly less‐likely to use firearms as a means of suicide (12/49 vs. 4,516/8414; *χ* ^2^ = 16.68, *P* < 0.001). When accounting for death age and sex, individuals with ASD were still significantly less likely to use firearms (OR: 0.33 [95% CI: 0.17–0.64]).

## Discussion

In this study, we used existing population‐wide suicide and ASD surveillance data to determine the incidence of suicide among individuals known to have a diagnosis of ASD in Utah over a 20‐year period. Through the use of unique data resources, we were able to conduct the first population‐based study of suicide in a U.S. statewide ASD population. Our study complements existing epidemiological work in Sweden [Hirvikoski et al., 2016]. Our results reveal lower incidence of suicide death for individuals with ASD than Hirvikoski et al.'s study, which may reflect differing methods of ascertainment of ASD status and/or actual differences in suicide rates. Rates could differ across countries for many reasons that are related to suicide rate differences in the general population including environmental factors [Holopainen, Helama, Björkenstam, & Partonen, 2013], sociocultural factors [Amitai & Apter, 2012], and service access and provision [Hester, 2017]. The incident rate identified in this study, which accounted for person‐time, suggested 32 suicides per 50,000 (6.4 per 10,000) person years in the ASD population during the 2013–2017 period.

Although, we observed slight increases in the incidence of suicide death in the ASD population across time, the rates were consistent with those in the general population except in the final time period. The diagnostic criteria for ASD has broadened over time, and ascertainment of accurate ASD diagnosis for those without ID has improved as well [CDC, 2018]. These changes could have affected the observed low incidences in the earlier time periods during which individuals with ASD without ID were likely to be under‐ascertained using a community‐based, administrative records approach, especially considering Hirvikoski et al.'s [2016] finding that suicide in individuals with autism occurred more commonly among those without ID.

The incidence estimates during the most recent time interval suggested suicide risk was higher in the ASD vs. the non‐ASD population; however, this difference was accounted for by females with ASD who died by suicide. Although there were no documented cases of suicide death among females with ASD during the first 15 years of the surveillance period, there was a sharp rise in the final time period (2013–2017) in which seven females with ASD completed suicide. The rate during this time period was equivalent to rates among males with and without ASD. There is evidence that women, especially those with higher intellectual functioning, are often diagnosed with ASD later in life, and some may never receive a formal diagnosis [Lai, Lombardo, Auyeung, Chakrabarti, & Baron‐Cohen, 2015]. Therefore, autistic females who died by suicide during the earlier years of this study may have been disproportionately less likely to receive an ASD diagnosis. In our analyses focused on young people during the 2013–2017 period, females with ASD were found to be over five times more likely to die by suicide than their non‐ASD peers, though the confidence intervals were notably large for this finding.

Our findings related to females with ASD in the most recent time period (2013–2017) are consistent with those of Hirvikoski et al. [2016]. Females with ASD have often been overlooked in research [Lai et al., 2015]; our study results provide further justification for enhancing focus on females. Some studies have shown that females with ASD may face a variety of challenges including sexual abuse, social difficulties, and conflicts between ASD traits and feminine identity [Bargiela, Steward, & Mandy, 2016]. Traumatic experiences and identity conflicts are known risk factors for suicide in the general population. Further, studies have shown that females may feel the need and have the ability to “camouflage” or cover‐up their autism symptoms [Bargiela et al., 2016], and one recent study suggested that these camouflaging behaviors are significantly associated with suicidality in ASD [Cassidy, Bradley, Shaw, & Baron‐Cohen, 2018].

Unemployment is common among individuals with ASD, and has been posited to relate to high rates of suicidal behavior [e.g., Pelton & Cassidy, 2017]. Thus, it is notable that approximately half (49%) of the individuals with ASD who completed suicide in our sample were listed on their death certificate to either have an occupation or be a student (an additional 40% had no data). These results may imply that individuals with ASD who are employed or enrolled in school are not necessarily at lower risk for suicide. Employment is an important priority for adults with ASD, but based on our findings, we note that placement in a job should not be viewed as *de facto* suicide prevention. Future studies should further examine employment status with more complete data, as well as other key factors such as job satisfaction, overall quality of life, and mental health.

When comparing individuals who died by suicide with and without ASD, we found that those with ASD were significantly younger in age when they died. This may be related to particular challenges faced by young people with ASD, such as identity formation and social difficulties, during adolescence and the transition to adulthood. Alternatively, this finding may be attributable to fewer older individuals with diagnoses of ASD resulting from increased measured prevalence of childhood ASD diagnosis over the last two decades (CDC, 2018). Emerging research on early mortality in ASD [e.g., Hirvikoski et al., 2016] may also contribute to explaining the observed age differences. We also looked specifically at risk among young people, and found that young people with ASD have over twice the risk of suicide than young people without ASD. Thus, there is a notable risk among youth with ASD; however, we caution interpretation that suicide is uniquely a problem for youth with ASD, and encourage future research to consider risks, potential causal factors, and prevention methods for older adults as well.

We also compared method of suicide between individuals with and without ASD. The groups did not differ in their use of violent and nonviolent methods. Prior research has suggested that individuals with ASD may attempt suicide with more lethal means [Kato et al., 2013] and may use particularly “bizarre acts” (Demirkaya, Tutkunkardas, & Mukaddes, 2016, p. 2923). In our sample, we did not see higher rates of violent methods, nor did we identify approaches that seemed unusual when comparing ASD versus non‐ASD suicide decedent groups. However, suicide decedents with ASD were significantly less likely than those without ASD to use firearms as a method of suicide. This difference was not accounted for by age at death or sex of the decedents, maintaining a three‐fold reduced risk of firearm use. In 2013, approximately 31.9% of adults in Utah owned a firearm, slightly exceeding the national average of 29.1% [Kalesan, Villarreal, Keyes, & Galea, 2016]. Although limiting firearms access is an important suicide prevention approach for any population [Lewiecki & Miller, 2013], the ASD group's use of non‐firearm suicide methods merits further consideration when developing suicide prevention efforts directed to this population.

### *Limitations & Future Directions*

Despite the strengths of population‐based research, there are limitations to this study. One limitation surrounds the challenge of complete ascertainment regarding both ASD and suicide classifications. Suicide can be difficult to determine, and determinations are made conservatively; medical examiners are reluctant to label a death as suicide if uncertain about the individual's intention to die [Rocket et al., 2014]. This may especially be the case among individuals with developmental disabilities such as ASD, for whom determining intent is no doubt difficult. Another limitation is that inadequate data on intellectual ability was available to examine the influence ID may have on suicide risk in individuals with ASD. ASD classifications may also be limited; not all individuals who may meet criteria for ASD have received a diagnosis, particularly among older individuals [Lai & Baron‐Cohen, 2015] and females [Loomes, Hull, & Mandy, 2017]. Further, we did not have access to additional data that could have enhanced the study, including data necessary to calculate suicide incidence rates in the non‐ASD population, to control for potential confounders in the incidence analyses, and to examine additional variables that may be important, such as socioeconomic status. Finally, case reports have described individuals who did not receive an ASD diagnosis until after a suicide attempt [Zahid & Upthegrove, 2017]. Suicide completion would preempt such opportunity for diagnosis. Therefore, our data may under‐represent the true incidence of suicide death in the ASD population.

Future population‐based studies should investigate suicidal ideation and suicide attempts in the ASD population as well as the influence of co‐occurring ID and psychiatric diagnoses on suicide risk associated with ASD. Additional research is needed on females with ASD in particular to determine potential sources of social and biological influences that could contribute to suicide risk. Further, while we recognize that there is evidence of multiple causes of premature mortality in ASD [Bilder et al., 2013; Hirvikoski et al., 2016; Mandell, 2018], few other causes of death affect such young individuals in the ASD population. Over half of the identified suicide death cases in the ASD population were individuals under age 30, and young people with ASD were found to be at greater risk than those without ASD. Therefore, better understanding of suicide risks among young people may particularly be important for future research.

## Conclusion

Using 20 years of surveillance data in Utah, we identified incidence of suicide among individuals with a diagnosis of ASD. Findings in the most recent time period suggest that individuals with ASD may be at higher risk for suicide. This risk is especially pronounced among females. Additional research is needed to apply population‐based methods to study suicide attempts and ideation in ASD and to improve understanding of the unique needs of females with ASD in regards to suicide risk.

## Conflicts of Interest

The authors have no conflicts of interest to declare.
